# Gut Microbiota Assembly and Host Phenotypic Variation: Core Adaptive Strategies of *Triplophysa yarkandensis* (Cypriniformes: Nemacheilidae) to Saline–Alkaline Stress

**DOI:** 10.3390/biology15090677

**Published:** 2026-04-25

**Authors:** Huijie Chen, Weicheng Wang, Xinyuan Ye, Li Feng, Mengbo Wang, Tingyu Xie, Daoquan Ren, Yong Song, Shengao Chen, Chi Zhang, Wentao Zhu

**Affiliations:** 1 College of Life Science and Technology, Tarim University, Alar 843300, China; chenhj@taru.edu.cn (H.C.); wwcyxh@126.com (W.W.); n3525177151@163.com (X.Y.); fengli15770896466@163.com (L.F.); wmb429322@163.com (M.W.); xxty242125@163.com (T.X.); rdqdky@126.com (D.R.); 120050013@taru.edu.cn (Y.S.); shengao@taru.edu.cn (S.C.); 2 Hubei Key Laboratory of Animal Nutrition and Feed Science, School of Animal Science and Nutritional Engineering, Wuhan Polytechnic University, Wuhan 430023, China; zhch@whpu.edu.cn

**Keywords:** *Triplophysa yarkandensis*, saline–alkaline adaptation, gut microbiome, morphology, host–microbe interaction

## Abstract

*Triplophysa yarkandensis* (Cypriniformes: Nemacheilidae), a rare endemic fish in the Tarim River Basin, Xinjiang, China, plays a pivotal role in maintaining the stability of plateau saline–alkaline aquatic ecosystems, yet its survival is increasingly threatened by habitat salinization. However, the multi-dimensional synergistic adaptation mechanisms linking its phenotypic variation, intestinal structure, and associated microbial communities to extreme saline–alkaline stress remain poorly understood. In this study, we innovatively integrated morphological/intestinal histological characterization, 16S rRNA gene sequencing, and microbial ecological analyses (co-occurrence networks and assembly processes) to systematically decode its adaptive strategies. Results revealed that *T. yarkandensis* exhibits a streamlined body shape, morphological variability, and elongated intestinal villi that may support locomotion and nutrient/ion uptake under osmotic stress. Its gut exerts a stringent selective filter, driving distinct differentiation between water and gut microbial communities—with gut-enriched core taxa (*Aurantimicrobium* and *Aestuariivirga*) and functional pathways (unsaturated fatty acid biosynthesis and ABC transporters) specialized for osmoregulation. Notably, the water microbial assembly is dominated by stochastic processes, while the gut assembly relies on host-driven deterministic selection, forming a habitat-specific adaptive pattern. These findings uncover the synergistic adaptation system of host phenotype and gut microbiota for survival in extreme saline–alkaline habitats, advancing our understanding of fish–microbe co-evolution in extreme ecosystems and providing critical theoretical support for the conservation of rare plateau fish, as well as guidance for the utilization of saline–alkaline water resources in aquaculture.

## Simple Summary

The rare fish *Triplophysa yarkandensis* is unique to the Tarim River Basin and lives in extreme saline–alkaline water environments, but how it adapts to such harsh living conditions remains unclear, which also hinders the effective protection of this precious endemic species. This study aimed to figure out the adaptive strategies of *T. yarkandensis* to saline–alkaline habitats and provide scientific support for its conservation. We found this fish has evolved a streamlined body shape and a specialized intestinal structure that helps it absorb nutrients and ions better in saline–alkaline water. Its intestinal tract can select specific microbes from the surrounding water to form a unique intestinal microbial community, which further assists the fish in regulating its body osmotic pressure and absorbing nutrients. The formation rules of water and intestinal microbes are also distinctly different. In conclusion, the synergy between the fish’s own body structure and its intestinal microbes is the key for it to survive in extreme saline–alkaline water. Our findings offer practical scientific guidance for the protection and artificial breeding of this rare fish and also help people understand how living things adapt to extreme natural environments.

## 1. Introduction

Extreme aquatic environments, such as saline–alkaline water bodies, represent a key selective pressure driving the adaptive evolution of fish species, with their adaptive mechanisms becoming a research hotspot in evolutionary ecology and environmental microbiology [1,2]. Saline–alkaline stress poses multiple physiological challenges to fish, including osmotic imbalance, nutritional limitation, and oxidative stress, which drive the formation of specialized morphological and microbial symbiotic characteristics in host fish [2,3,4]. The Tarim River Basin in Xinjiang, China, is a typical area with widespread saline–alkaline water ecosystems, and its endemic fish species have evolved unique adaptive strategies to cope with the local extreme aquatic environment after long-term natural selection. *Triplophysa yarkandensis* (Day, 1877), a rare endemic loach species in this region and a dominant fish in the Kizil River and other typical saline–alkaline water bodies of the Tarim River Basin, plays an irreplaceable ecological role in maintaining the stability of the aquatic food web, regulating material cycling, and indicating the health status of the aquatic ecosystem [5,6]. In recent years, however, the wild population of *T. yarkandensis* has declined sharply due to the intensification of water body saline–alkalization, habitat fragmentation, and human interference. Clarifying its environmental adaptation mechanisms is not only crucial for the protection and restoration of this rare germplasm resource but also provides a typical case for understanding the adaptive evolution of fish in plateau saline–alkaline water bodies worldwide.

Morphological plasticity is the primary adaptive response of fish to extreme environmental stress, and it is the material basis for host survival and reproduction under adverse conditions [7,8]. For fish in saline–alkaline environments, streamlined body shape and phenotypic variation in key morphological parameters (e.g., body height and head length) can optimize their swimming efficiency in flowing saline–alkaline water and help them adapt to the foraging behavior of local food resources [2,9,10]. As the core organ for nutrient absorption and osmotic regulation in fish, the intestinal tract’s histological structure (e.g., villus height, density, and muscular layer thickness) is closely linked to the host’s nutritional utilization efficiency and osmotic balance maintenance [11]. Elongated intestinal villi can expand the mucosal surface area, thereby enhancing the absorption of nutrients and ions, which is a typical histological adaptation of fish to cope with nutritional limitation and osmotic stress in saline–alkaline habitats [12]. Although previous studies have conducted preliminary morphological descriptions of *T. yarkandensis*, systematic quantitative analysis of its key morphological parameters and intestinal histological characteristics, as well as the functional correlation between these traits and saline–alkaline adaptation, remains unclear, which limits the understanding of its phenotypic adaptive basis.

The gut microbiota, as a “second genome” of fish, participates in a variety of host physiological processes including nutrient metabolism, immune defense, and osmotic regulation through complex interactions with the host and forms a tight symbiotic system with the host to jointly respond to environmental stress [13]. The assembly of fish gut microbiota is mainly shaped by the host’s selective filtration of environmental microorganisms, and the water microbiota, as the most important source of gut microbiota, has a profound impact on the structure and function of the host’s gut microbial community [14]. In saline–alkaline water bodies, the environmental microbial community is characterized by high salinity tolerance and specific metabolic functions, and the host fish can screen and enrich functional microbial taxa adapted to the intestinal niche from the complex environmental microbial pool, forming a gut microbiota with host-specific and habitat-specific characteristics [14,15]. For plateau fish such as *schizothoracids*, studies have found that their gut core microbiota is closely involved in lipid metabolism and ion transport and plays a key role in the host’s adaptation to high-altitude and saline–alkaline environments [6]. However, for *T. yarkandensis*, the structural and diversity differences between its water and gut microbial communities, the assembly rules of gut microbiota, and the functional characteristics of core microbial taxa in saline–alkaline habitats have not been reported yet, and the interaction mechanism between water and gut microbial communities remains to be further explored.

The synergistic adaptation between the host’s phenotypic/histological traits and gut microbiota is an important evolutionary strategy for fish to cope with extreme environmental stress, and the correlation between microbial community structure and host phenotypic characteristics has become a key research direction in the field of fish–microbe symbiosis [16]. Environmental stress can drive the co-evolution of the host’s phenotypic variation and gut microbiota functional specialization: the host’s specific morphological and histological traits provide a suitable niche for the colonization and reproduction of functional microbiota, while the gut microbiota can regulate the host’s nutrient absorption and tissue development and further shape the host’s phenotypic characteristics [17]. In freshwater and marine fish, studies have confirmed that the relative abundance of core gut microbial taxa is significantly associated with the host’s intestinal villus height, body length, and other traits [16,17,18,19]. However, for *T. yarkandensis* in plateau saline–alkaline water bodies, the quantitative correlation between its gut microbial community and host morphological/intestinal histological traits has not been revealed, which makes it impossible to clarify the synergistic adaptation mechanism of the “host–microbiota” complex system to saline–alkaline stress.

In addition, the existing research on *T. yarkandensis* is mostly limited to basic resource investigation and simple biological characteristic description, and there is a lack of integrated analysis of multiple physiological and ecological characteristics. The single research dimension makes it difficult to comprehensively and systematically reveal its environmental adaptation mechanism. In view of the above research gaps, this study takes *T. yarkandensis* in the saline–alkaline water body of the Tarim River Basin as the research object and combines morphological measurement, intestinal histological staining, 16S rRNA gene high-throughput sequencing, and bioinformatics analysis to carry out the following research: (1) systematically analyze the morphological phenotypic variation and intestinal histological structural characteristics of *T. yarkandensis*; (2) reveal the structural, diversity and compositional differences between water and gut microbial communities, and identify the core and differential microbial taxa of the two habitats; (3) predict the functional characteristics of water and gut microbial communities and clarify their functional differentiation in response to saline–alkaline stress; (4) explore the quantitative correlation between gut core microbial taxa and host morphological/intestinal histological traits. This study is expected to reveal the phenotypic and microbial adaptive basis of *T. yarkandensis* to saline–alkaline environments from the perspective of the “host–microbiota” synergy, enrich the theoretical system of fish environmental adaptation in plateau saline–alkaline water bodies, and provide important theoretical support for the germplasm resource protection, artificial breeding optimization and saline–alkaline water fish resource utilization of *T. yarkandensis*.

## 2. Materials and Methods

### 2.1. Experimental Fish and Ethical Statement

Healthy adult *T. yarkandensis* individuals (intact body surface; normal swimming/feeding behavior; no hemorrhage, ulceration, parasite infestation or visible abnormalities; body length: 8.0–15.0 cm; body weight: 10.0–25.0 g; *n* ≥ 50) were collected from the Kizil River (Tarim River Basin, Xinjiang Uygur Autonomous Region, China), a typical saline–alkaline water habitat of this species. The environmental parameters of the sampling site were measured in situ using a multi-parameter water quality analyzer (YSI ProDSS, Yellow Springs, OH, USA): water temperature 18.0–22.0 °C, pH 8.5–9.2, salinity 3.2–4.5‰, dissolved oxygen (DO) 6.0–7.5 mg L^−1^, and total alkalinity 12.0–15.5 mmol L^−1^.

Prior to the experiment, fish were acclimated in three fully independent, parallel 300 L recirculating aquaculture tanks (stocking density: 16–17 fish per tank; ≈0.05 fish per liter) under laboratory conditions that strictly mimicked all key physicochemical parameters of the Kizil River habitat (water parameters consistent with the sampling site) for 2 weeks. During acclimation, fish were fed a commercial fish feed once daily at 1% of body weight, with 30% water renewal every 2 days and continuous aeration to maintain DO > 6.0 mg L^−1^. The photoperiod was set to 12 h light:12 h dark. All fish were fasted for 24 h before sample collection, which is a standardized protocol widely used in fish intestinal research to effectively avoid feed interference with intestinal microbiota and histological structure.

All experimental procedures involving live fish were approved by the Animal Ethics Committee of Tarim University (Approval No.: PA20250326088). Fish were anesthetized with MS-222 (tricaine methanesulfonate, 100 mg L^−1^) prior to all sampling operations to minimize pain, in strict accordance with the Guidelines for the Care and Use of Laboratory Animals in China.

### 2.2. Sample Collection

All sampling was performed in a sterile ultra-clean bench with three biological replicates for water and gut microbial samples, and morphological/histological analyses using sufficient individuals for statistical validity. Rearing water samples (500 mL per replicate) were collected in sterile polypropylene bottles, transported on ice, and processed within 2 h. Anesthetized fish were blotted dry, and key morphological traits were measured with a digital vernier caliper (0.01 mm precision) and electronic balance (0.01 g precision). Fish were then dissected with sterile instruments to isolate the entire intestinal tract, with mesentery and adipose tissue removed. The middle intestine was cut into 5 mm segments and fixed in 4% paraformaldehyde (PFA, pH 7.4, Biosharp, Taizhou, Jiangsu, China) at 4 °C for 24 h for histological analysis (*n* = 10 sections from 5 individuals). The mid-intestine was chosen for analysis because it is the primary site of nutrient absorption, ion exchange, and gut microbiota colonization in fish and is highly representative of the host’s adaptive traits to saline–alkaline stress. Intestinal contents were gently squeezed out, transferred to sterile 2 mL centrifuge tubes, snap-frozen in liquid nitrogen, and stored at −80 °C for microbial DNA extraction. Three biological replicates were prepared for intestinal content samples, and each replicate pool contained contents from 5 fish to reduce individual variation. All surgical instruments were sterilized by autoclaving (121 °C, 30 min) and 75% alcohol soaking to avoid cross-contamination. All sampling steps were performed in accordance with the standard operational protocols for fish sampling widely used in fisheries biology research.

### 2.3. Morphological Trait Analysis

Ten key morphological traits related to saline–alkaline adaptation were measured for 50 *T. yarkandensis* individuals: body length, body height, head length, caudal peduncle length, snout length, mouth gape length, mouth gape width, eye diameter, barbel length, and body weight. Each trait was measured in three technical replicates per individual, and all measurements were performed by a single trained operator to ensure consistency. Measurement data were recorded in Microsoft Excel 2021, and descriptive statistical analysis (mean ± standard error of the mean, SEM) was conducted to characterize population phenotypic variation and distribution. Phenotypic variation in traits was identified based on interquartile range (IQR), providing a basis for subsequent correlation analysis with intestinal microbiota. All morphological measurements were performed in accordance with standard operational protocols for fish morphological analysis widely used in fisheries biology research.

### 2.4. Intestinal Histological Preparation and Observation

Intestinal tissue fixed in 4% PFA was processed for paraffin embedding via gradient ethanol dehydration, xylene transparency, molten paraffin impregnation (58–60 °C, 2 h), and embedding into solid blocks. Blocks were cut into 5 μm serial sections with a Leica RM2235 rotary microtome (Leica Biosystems Nussloch GmbH, Nussloch, Baden-Württemberg, Germany), mounted on poly-L-lysine-coated slides, and dried at 60 °C for 2 h. Sections were subjected to standard hematoxylin and eosin (HE) staining: deparaffinization, gradient rehydration, hematoxylin staining (5 min), 1% hydrochloric acid ethanol differentiation (30 s), tap water bluing (15 min), eosin staining (2 min), followed by gradient dehydration, xylene transparency, and neutral balsam mounting. Stained sections were observed under an Olympus BX53 light microscope (100× and 400× magnifications, Olympus Corporation, Tokyo, Japan), with representative fields captured by an Olympus DP74 digital camera (Olympus Corporation, Tokyo, Japan). Image-Pro Plus 6.0 software was used for quantitative analysis of villus height (VH), villus width (VW), and muscular thickness (MT); ten intact intestinal villi were measured in each of two non-overlapping fields for each single section, and the mean value was used as the final individual data to ensure accuracy.

### 2.5. Microbial DNA Extraction and Quality Detection

Rearing water samples were filtered through 0.22 μm sterile cellulose acetate filter membranes to collect microbial biomass, and genomic DNA was extracted with the Omega Soil DNA Kit (Omega Bio-tek, Inc., Norcross, GA, USA). Intestinal content DNA (0.2 g per sample) was extracted using the Omega Stool DNA Kit (Omega Bio-tek, Inc., Norcross, GA, USA) to reduce host DNA and food residue interference, with three technical replicates per sample whose extracts were mixed for subsequent experiments. DNA concentration and purity were determined with a NanoDrop 2000 UV-Vis spectrophotometer (qualified as OD_260_/OD_280_ = 1.8–2.0, Thermo Fisher Scientific, Wilmington, DE, USA), and integrity was verified by 1% agarose gel electrophoresis (120 V, 20 min) with a Bio-Rad Gel Doc XR+ (Bio-Rad Laboratories, Inc., Hercules, CA, USA) imaging system. Qualified DNA was diluted to 10 ng μL^−1^ with sterile TE buffer (pH 8.0) and stored at −80 °C for PCR amplification and sequencing.

### 2.6. Bacterial 16S rRNA Gene Amplification and High-Throughput Sequencing

The V3-V4 hypervariable region of the bacterial 16S rRNA gene was amplified with universal primers 338F (5′-ACTCCTACGGGAGGCAGCAG-3′) and 806R (5′-GGACTACHVGGGTWTCTAAT-3′), with the forward primer labeled with a unique 8 bp barcode for each sample. PCR amplification was performed in a 25 μL system containing 12.5 μL of 2× Taq PCR MasterMix, 1 μL of each primer (10 μmol L^−1^), 2 μL of DNA template (10 ng μL^−1^), and 8.5 μL of sterile ddH_2_O. The amplification program was: pre-denaturation at 95 °C for 3 min; 30 cycles of 95 °C (30 s), 55 °C (30 s), and 72 °C (45 s); final extension at 72 °C for 10 min; and storage at 4 °C. PCR products were detected by 1.5% agarose gel electrophoresis, target bands (≈468 bp) were excised and purified with AMPure XP magnetic beads (Beckman Coulter, Inc., Indianapolis, IN, USA), and concentration was quantified with a Qubit 3.0 Fluorometer (Thermo Fisher Scientific, Waltham, MA, USA). Equal amounts of purified products (100 ng per sample) were mixed to construct a sequencing library, whose quality was verified with an Agilent 2100 Bioanalyzer (Agilent Technologies, Inc., Santa Clara, CA, USA). Qualified libraries were sequenced on the Illumina MiSeq PE300 platform by Novogene Bioinformatics Technology Co., Ltd. (Beijing, China).

### 2.7. Bioinformatics Analysis of Microbial Communities

Raw paired-end reads were demultiplexed by barcodes, and low-quality sequences were filtered with Trimmomatic v0.39 (Phred score < 20; length < 200 bp; ambiguous bases and primer mismatches removed). Clean reads were merged into contigs with FLASH v1.2.11 (overlap ≥ 10 bp; mismatch rate < 0.2%), chimeric sequences were removed with UCHIME v4.2 against the Silva 138 database, and singleton Amplicon Sequence Variants (ASVs) were excluded to obtain high-quality ASVs. Taxonomic annotation of ASVs was performed with RDP Classifier v2.13 (confidence threshold 0.7) against the Silva 138 database, with unclassified and archaeal sequences removed. Alpha diversity indices (Chao1, Shannon, Pielou’s evenness, Observed species, and Good’s coverage) were calculated with the vegan package in R 4.3.1, and rarefaction curves were plotted to verify sequencing depth. Beta diversity was analyzed via Bray–Curtis dissimilarity matrix construction, principal coordinate analysis (PCoA), hierarchical clustering (UPGMA), and Analysis of Similarities (ANOSIM) for group difference significance. Microbial community composition at the phylum, genus, and species levels was calculated, with low-abundance taxa (<0.5%) grouped as “Others”, and visualized via bar plots, bubble plots, and heatmaps with ggplot2 v3.4.4. Linear Discriminant Analysis Effect Size (LEfSe) was used to identify differential taxa (LDA score > 3.0, *p* < 0.05), and Venn diagrams were constructed to analyze unique and shared ASVs. Co-occurrence network analysis of core genera (>0.5%) was performed via Spearman’s correlation matrix (*p* < 0.05, |*r*| > 0.6), with networks constructed and visualized in Cytoscape v3.9.1 (ForceAtlas2 layout) and topological parameters calculated to identify core nodes and potential microbial transmission taxa. Microbial functional potential was predicted with PICRUSt2 v2.5.0, with 16S rRNA gene copy number normalization, Kyoto Encyclopedia of Genes and Genomes (KEGG) ortholog annotation, and LEfSe used to identify differential KEGG pathways (LDA score > 2.0, *p* < 0.05).

### 2.8. Statistical Analysis

All statistical analyses were performed with R 4.3.1, GraphPad Prism 9.0, and SPSS 26.0, with data presented as mean ± SD and significance set at *p* < 0.05. Shapiro–Wilk and Levene’s tests were used to verify data normality and variance homogeneity, respectively. Independent-samples *t*-tests were used for normally distributed data with homogeneous variances and Wilcoxon rank-sum tests for non-normally distributed data. Pearson correlation analysis was used to explore linear correlations between gut core microbial genera and host morphological/histological traits and Mantel tests (999 permutations) to analyze overall correlations between gut microbial community composition and host phenotypic traits. All figures were drawn with R v4.3.1 (ggplot2 v3.4.4, vegan v2.6-4, and pheatmap v1.0.12) and GraphPad Prism 9.0 at 300 dpi (TIFF/PNG format). Data are expressed as mean ± standard deviation (SD) to characterize phenotypic variation within the natural population. Between-group comparisons and mechanistic validation were not performed, as this is a descriptive study without experimental treatment groups.

## 3. Results

### 3.1. Morphological and Intestinal Histological Characterization of T. yarkandensis

**Figure 1:**
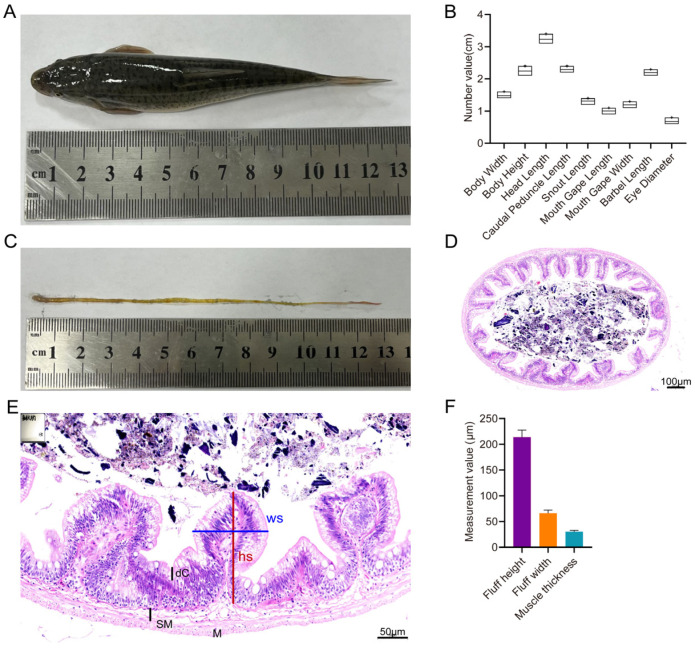
Morphological and intestinal histological characteristics of *T. yarkandensis*. (**A**) Representative photograph of adult *T. yarkandensis* (scale bar = 1 cm). (**B**) Frequency distribution of key morphological traits in the sampled population (*n* = 50). (**C**) Schematic of intestinal tract segmentation. (**D**) H&E-stained cross-section of middle intestine (scale bar = 200 μm; SM, submucosa; M, muscularis). (**E**) High-magnification view of intestinal mucosa (scale bar = 50 μm; WS, villus core). (**F**) Quantitative measurement of intestinal structural traits (*n* = 9, mean ± SD).

Adult *T. yarkandensis* exhibited a streamlined body shape (Figure 1A). Quantitative analysis of key morphological traits across the sampled population revealed distinct inter-individual variation (Figure 1B). The intestinal tract of this species exhibited an elongated morphological structure (Figure 1C). Histological observation revealed clear and intact intestinal villus structures with distinct morphological features (Figure 1D–F). Hematoxylin and eosin (H&E)-stained sections of the middle intestine displayed a well-structured intestinal wall with clearly differentiated submucosa and muscularis layers, as well as densely packed villi projecting into the lumen (Figure 1D). High-magnification imaging further resolved the detailed mucosal architecture of the villi, including the villus core and measurable height and width parameters (Figure 1E). The mucosa contained scattered goblet cells and sparse intraepithelial lymphocytes. The lamina propria consisted of loose connective tissue, and the submucosa was clearly demarcated with a normal structure. Morphometric quantification of intestinal structural traits confirmed villus height as the most prominent feature (mean ± SD, ~210 μm), which was higher than villus width and muscular thickness (Figure 1F), reflecting a distinct structural feature of the intestinal mucosa.

### 3.2. Microbial Community Structure and Diversity in Rearing Water and Gut Contents

**Figure 2:**
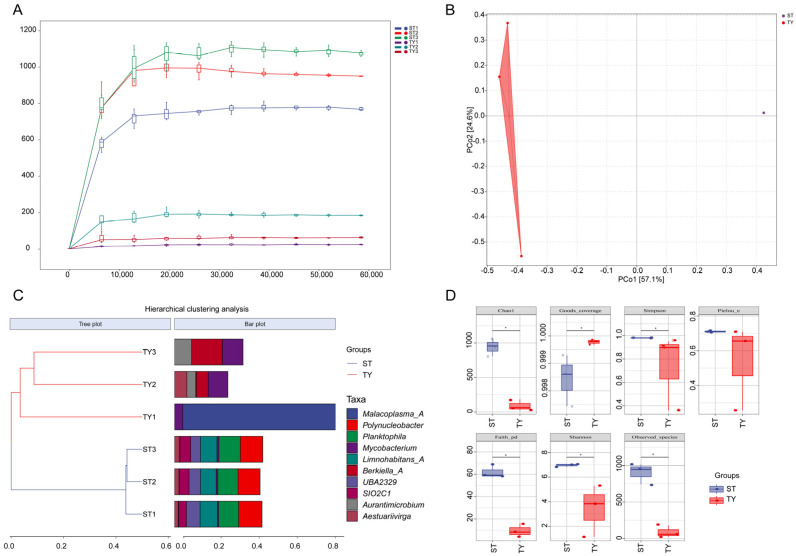
Microbial community diversity and structure in rearing water (ST) and gut contents (TY). (**A**) Rarefaction curves of observed species for ST and TY samples. (**B**) PCoA based on Bray–Curtis dissimilarity (PCo1 = 57.1%; PCo2 = 24.6%). (**C**) Hierarchical clustering (Bray–Curtis) with associated bar plot of dominant taxa relative abundance. (**D**) Boxplots of alpha diversity indices; * *p* < 0.05 (Wilcoxon rank-sum test). *n* = 3 per group. All samples represent fully independent biological replicates (*n* = 3 per group). The high clustering of ST replicates reflects highly stable and homogeneous water microbial communities under strictly controlled acclimation conditions.

Illumina MiSeq sequencing of the 16S rRNA gene V3–V4 region for three rearing water (ST) and three gut content (TY) samples generated a total of 618,496 high-quality sequences after quality filtering, denoising, chimera removal and singleton exclusion, with the detailed sequencing data and species composition of each sample summarized in Table 1. Taxonomic profiling showed a stark contraction in taxonomic complexity from ST to TY samples, with ST samples harboring 26–34 orders and 333–414 species per sample while TY samples only contained 0–8 orders and 12–44 species. Rarefaction curves of observed species for all samples reached saturation (Figure 2A), and Good’s coverage exceeded 0.99 for both ST and TY groups, confirming adequate sequencing depth for subsequent analyses. Principal coordinate analysis (PCoA) based on Bray–Curtis dissimilarity revealed clear compositional segregation between ST and TY microbial communities, with the first two axes collectively explaining 81.7% of the total variance (Figure 2B). This habitat-based partitioning was further corroborated by hierarchical clustering analysis, where samples grouped strictly by their source with distinct taxonomic profiles in the associated bar plot (Figure 2C). Alpha diversity analysis showed ST samples exhibited significantly higher species richness (Chao1), diversity (Shannon) and evenness (Pielou’s) compared to TY samples (*p* < 0.001), with all alpha diversity indices displaying obvious intergroup differences in boxplot analysis (Figure 2D), collectively demonstrating a strong host-mediated filtering effect of the *T. yarkandensis* gut on environmental microbial assemblages.

### 3.3. Habitat-Specific Structuring of Microbial Taxonomic Assemblages

**Figure 3:**
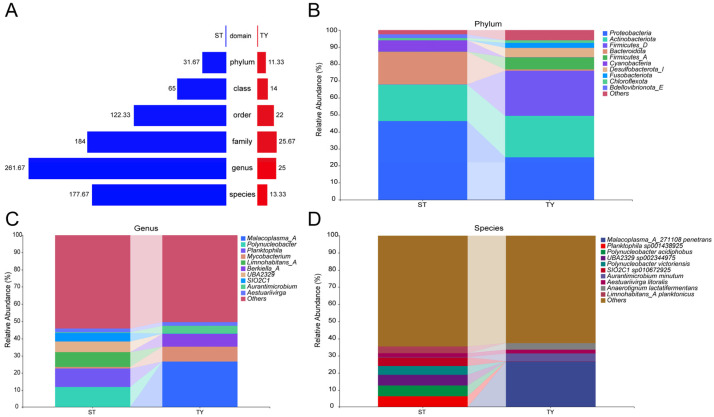
Taxonomic composition of ST and TY microbial communities across multiple ranks. (**A**) Bar plot of taxon numbers at each phylogenetic level (mean ± SD). (**B**) Relative abundance of dominant bacterial phyla. (**C**) Relative abundance of dominant bacterial genera (low-abundance taxa as Others). (**D**) Relative abundance of dominant bacterial species. *n* = 3 per group.

**Figure 4:**
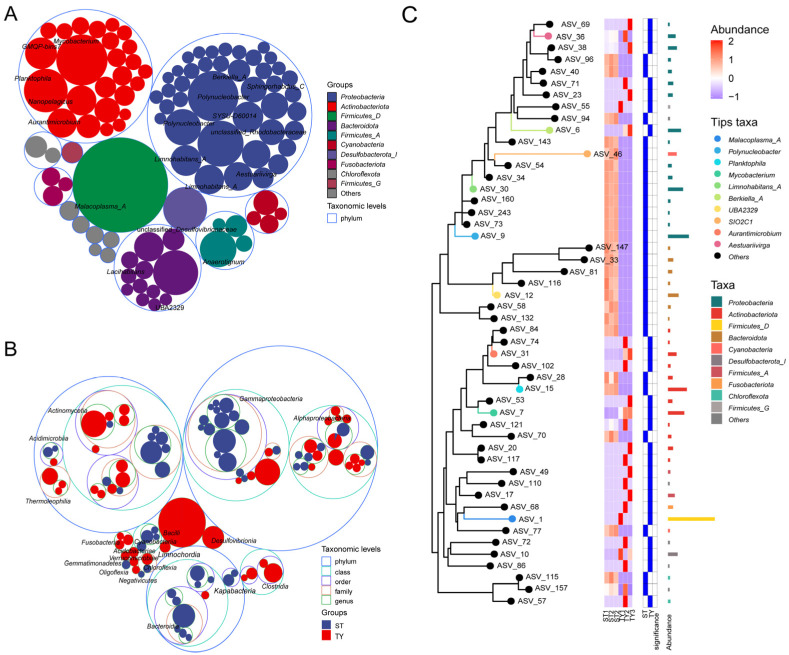
Distribution and phylogeny of microbial taxa in ST and TY habitats. (**A**,**B**) Bubble plots of phylum (**A**)- and genus (**B**)-level taxa (bubble size = mean relative abundance; color intensity = prevalence). (**C**) Phylogenetic tree of ASVs with paired heatmap of log-transformed relative abundance (branch color = phylum; tip color = habitat enrichment: blue = ST, red = TY).

Multi-level taxonomic compositional analysis across the full phylogenetic hierarchy (Domain to Species) revealed a consistent pattern of greater diversity in ST samples relative to TY samples, with ST harboring on average 29.7 phyla, 65 classes and 261.7 genera while TY only contained 11.3 phyla, 14 classes and 25 genera (Figure 3A). At the phylum level, ST communities were characterized by a saline–alkaline water typical profile dominated by Proteobacteria (~45%), *Actinobacteriota* (~25%) and *Bacteroidota* (~15%), whereas TY communities exhibited a restructured profile with Proteobacteria remaining prevalent (~30%) and *Bacteroidota* (~25%) and *Firmicutes* (~15%) being substantially enriched (Figure 3B). Genus-level analysis showed the ST microbiota comprised a diverse suite of dominant taxa including *Malacoplasma_A*, *Polynucleobacter* and *Mycobacterium*, while the TY microbiota was overwhelmingly dominated by a small number of genera such as *Aurantimicrobium* and *Aestuariivirga*, with most sequences grouped as “Others” (Figure 3C), and this low-richness pattern was consistent at the species level (Figure 3D). Bubble plot visualization further emphasized the disparities in taxon prevalence and abundance between the two habitats: ST samples displayed numerous, large bubbles representing ubiquitous and abundant phyla (Figure 4A) and genera (Figure 4B), while TY samples showed fewer, smaller bubbles, confirming the limited taxonomic representation in the intestinal environment. The phylogenetic tree of amplicon sequence variants (ASVs) with a paired heatmap further revealed clear habitat enrichment of microbial taxa, with ASVs colored blue for ST and red for TY showing distinct clustering at the genus and phylum levels (Figure 4C).

### 3.4. Core and Differential Taxa and Functional Potentials Between Water and Gut Microbial Communities

**Figure 5:**
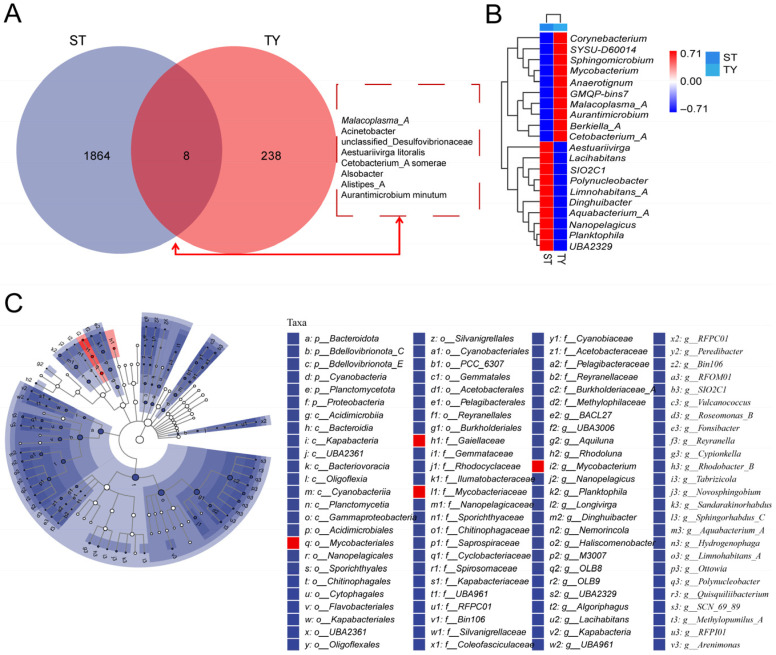
Shared and differential microbial taxa between ST and TY communities. (**A**) Venn diagram of ASVs in ST and TY samples. (**B**) LEfSe cladogram of differentially abundant taxa (LDA score > 3; blue = ST-enriched, red = TY-enriched). (**C**) Circular phylogenetic tree of habitat-specific taxa (branch color = habitat preference: blue = ST, red = TY).

**Figure 6:**
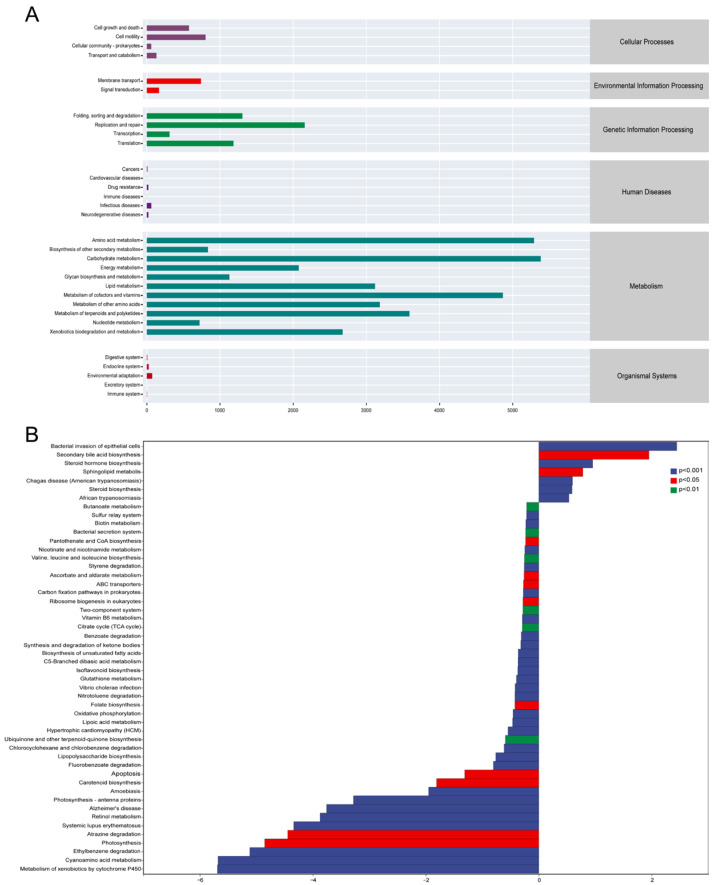
Functional potential of ST and TY microbial communities. (**A**) Relative abundance of KEGG pathways at the category level (mean ± SD). (**B**) LEfSe analysis of differentially enriched KEGG pathways (LDA score > 2; blue = ST-specific, red = TY-specific).

Amplicon sequence variant (ASV)-level analysis revealed strong habitat specificity of the microbial communities, with ST samples containing 1864 unique ASVs, TY samples hosting 238 unique ASVs, and only eight ASVs shared between the two habitats (Figure 5A). Linear Discriminant Analysis Effect Size (LEfSe) identified taxa with significant habitat preferences (LDA score > 3), with ST-enriched taxa including *Corynebacterium*, *Sphingobacterium* and *Mycobacterium* and TY-enriched taxa featuring *Aurantimicrobium*, *Aestuariivirga* and *Limnohabitans_A* (Figure 5B). A circular phylogenetic tree contextualized these patterns evolutionarily, showing ST-associated taxa predominantly affiliated with *Bacteroidota*, *Desulfovibrionota_E* and *Cyanobacteria* while TY-enriched taxa clustered within *Mycobacteriales* and *Gordoniaceae* (Figure 5C), reflecting divergent selection pressures in aquatic and intestinal habitats. PICRUSt2-based KEGG-predicted functional potential revealed metabolic pathways dominated both communities with obvious quantitative differences (Figure 6A): ST samples were enriched in environmentally relevant pathways such as xenobiotics biodegradation, while TY samples exhibited predicted heightened activity in genetic information processing pathways. LEfSe analysis of KEGG pathways (LDA score > 2) identified ST-specific pathways including bacterial invasion of epithelial cells and starch and sucrose metabolism, and TY-enriched pathways featuring biosynthesis of unsaturated fatty acids and ABC transporters (Figure 6B), which are critical for membrane homeostasis and nutrient uptake in the osmoregulatory gut environment, demonstrating functional complementarity between the taxonomically distinct ST and TY microbiomes.

### 3.5. Co-Occurrence Association Network and Topological Characteristics of Water and Gut Microbial Communities

**Figure 7:**
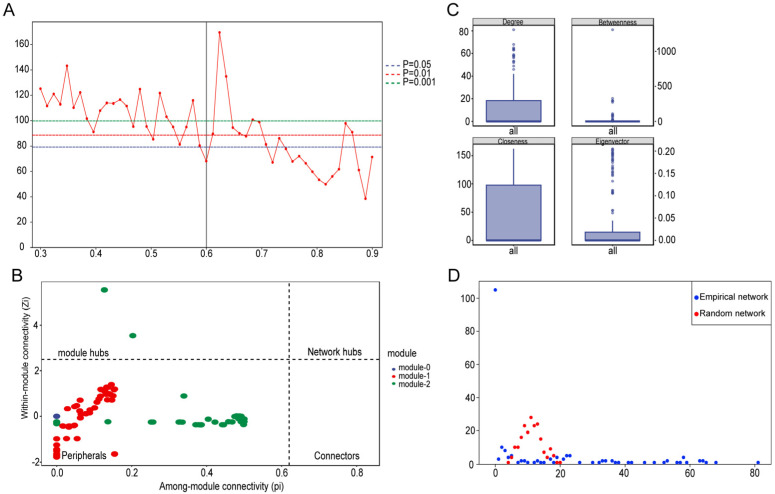
Co-occurrence network and topological characteristics of ST and TY microbial communities. (**A**) RMT threshold selection curve (optimal threshold = 0.85, red dashed line). (**B**) Co-occurrence network (node size = degree value; node color = habitat: blue = ST, red = TY, purple = shared; edge color = correlation: blue = positive, red = negative). (**C**) Comparison of core topological indices (mean ± SD; one-way ANOVA). (**D**) Degree distribution curves with power-law fitting (ST: R^2^ = 0.86; TY: R^2^ = 0.83).

**Figure A1:**
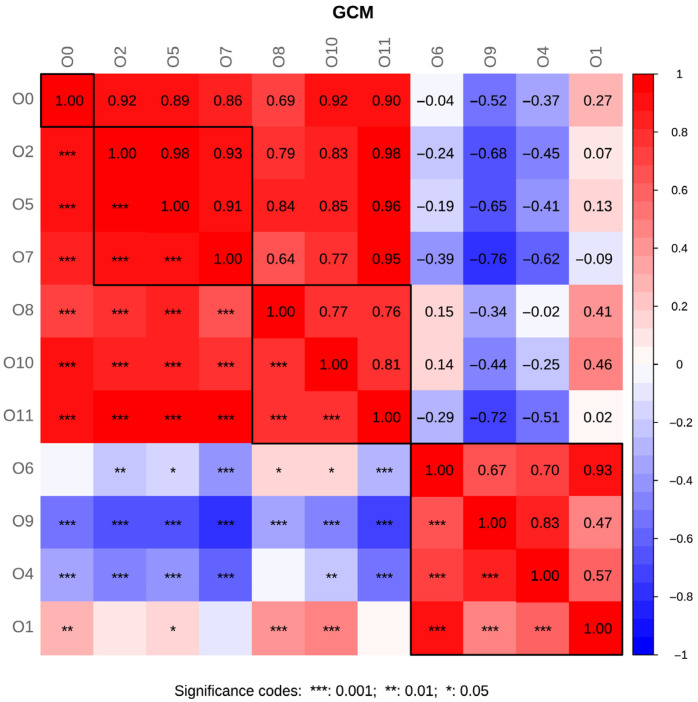
Gene co-expression model (GCM) similarity matrix heatmap of microbial taxa in rearing water (ST) and gut content (TY) groups (genus-level analysis, *n* = 3 per group). X and Y axes represent the microbial samples (O1–O11), with the color gradient indicating pairwise similarity coefficients (ranging from −1 to 1; red = high positive similarity, blue = high negative similarity). Significance codes: *** *p* < 0.001; ** *p* < 0.01; * *p* < 0.05. The heatmap reveals higher intragroup similarity and lower intergroup similarity between ST and TY microbial communities, providing a reliable biological basis for subsequent co-occurrence network analysis.

Gene co-expression model (GCM) similarity matrix heatmap analysis of all microbial taxa revealed obvious similarity differentiation between ST and TY groups, with higher intragroup similarity and lower intergroup similarity (Figure A1), providing a reliable biological basis for co-occurrence network construction. Based on Random Matrix Theory (RMT), the inflection point of the average degree-similarity threshold curve was used to determine the optimal similarity threshold of 0.85 for network construction (Figure 7A). The constructed co-occurrence network identified core hub nodes (top 10% of degree value) with *Polynucleobacter* as the dominant hub for ST and *Aurantimicrobium* for TY, where node size was positively correlated with degree value, edge color denoted correlation type and edge thickness reflected the correlation coefficient (Figure 7B). Quantitative analysis of four core topological indices showed significant intergroup differences among ST, TY and shared nodes (*p* < 0.05): ST nodes had remarkably higher Degree and Eigenvector Centrality, while TY nodes exhibited relatively higher Betweenness Centrality (Figure 7C). Degree distribution curves of the ST and TY microbial co-occurrence networks both conformed to the power-law distribution with high fitting degrees (ST: R^2^ = 0.86; TY: R^2^ = 0.83) (Figure 7D), presenting typical scale-free topological characteristics and indicating strong resistance to environmental disturbances of the microbial communities.

### 3.6. Ecological Niche Differentiation and Community Assembly Process of Water and Gut Microbial Communities

**Figure 8:**
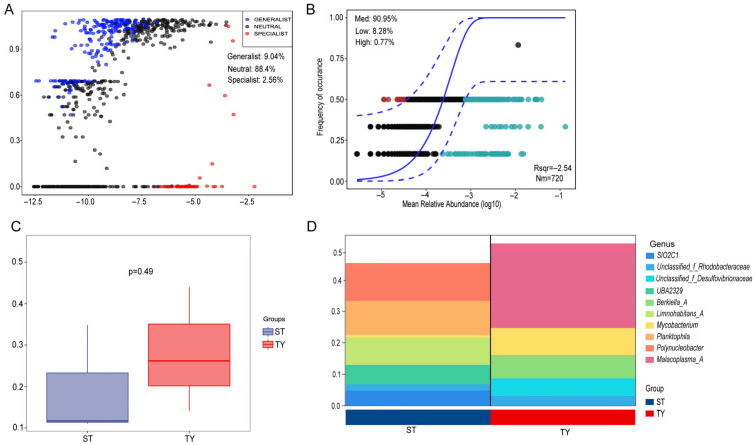
Ecological niche differentiation and community assembly of ST and TY microbial communities. (**A**) Scatter plot of Levins’ niche breadth and Schoener’s niche overlap (dot size = relative abundance; blue = ST, red = TY). (**B**) Neutral Community Model analysis (black line = fitting curve; gray shadow = 95% CI; ST: R^2^ = 0.45, m = 0.09; TY: R^2^ = 0.23, m = 0.04). (**C**) NST analysis (dashed line = 0.5; *p* < 0.01; Wilcoxon rank-sum test). (**D**) Top 10 taxa with the highest contribution degree (blue = ST, red = TY).

Ecological niche analysis based on Levins’ niche breadth and Schoener’s niche overlap revealed divergent resource utilization strategies between ST and TY microbial taxa (Figure 8A): ST taxa featured broader niche breadth and higher niche overlap with most clustered in the high-breadth and high-overlap region, while TY taxa exhibited narrow niche breadth and low niche overlap, a typical host-mediated niche specialization pattern. Neutral Community Model (NCM) analysis showed the ST community had a higher fitting degree to the neutral model (R^2^ = 0.45, m = 0.09) with more taxa distributed within the 95% confidence interval, whereas the TY community had a much lower fitting degree (R^2^ = 0.23, m = 0.04) with most taxa outside the interval (Figure 8B), indicating divergent assembly processes between the two habitats. Normalized Stochasticity Ratio (NST) analysis further quantified this difference, with the ST community having a significantly higher NST value of 0.71 (NST > 0.5) and the TY community a markedly lower NST value of 0.32 (NST < 0.5) (*p* < 0.01) (Figure 8C), confirming stochastic processes dominated ST microbial assembly while deterministic processes drove TY microbial assembly. Species contribution degree analysis identified the core taxa driving community assembly and niche differentiation: the top three contributing taxa for ST were *Polynucleobacter*, *Mycobacterium* and *Limnohabitans_A*, while for TY they were *Aurantimicrobium*, *Aestuariivirga* and unclassified *Rhodobacteraceae* (Figure 8D), which were consistent with the dominant taxa and core hub nodes identified in previous analyses.

## 4. Discussion

This study integrated morphological and intestinal histological characterization, 16S rRNA gene high-throughput sequencing, and microbial ecological analysis to systematically elucidate the adaptive strategies of *T. yarkandensis*—an endemic rare fish species in the saline–alkaline water of the Tarim River Basin—to extreme saline–alkaline habitats. As a typical representative of plateau loaches in arid northwest China, this species has evolved unique survival mechanisms under long-term natural selection, yet its saline–alkaline adaptation mechanisms from the perspective of host–microbiota synergy remain poorly understood. Saline–alkaline stress is a key abiotic factor driving the adaptive evolution of aquatic organisms, imposing severe osmotic imbalance, nutritional limitation, and physiological stress on fish [20], and the interaction between host phenotypic variation and associated microbial community characteristics has become a core research focus in the field of aquatic organism environmental adaptation [1,21]. This study fills the research gap in the integrated analysis of phenotypic and microbial adaptive traits of *T. yarkandensis* and provides a novel experimental case for understanding the adaptation mechanisms of fish in plateau saline–alkaline aquatic ecosystems.

The morphological and intestinal histological traits of *T. yarkandensis* exhibited obvious adaptive characteristics matching the saline–alkaline flowing water habitat, which conforms to the phenotypic evolution law of fish in extreme aquatic environments [6,22]. The streamlined body shape of adult fish is a typical morphological adaptation to reduce swimming resistance in flowing water, a common adaptive trait of riverine fish facing hydrodynamic stress [23,24]. The high phenotypic variation in body height and head length enables the species to adjust its foraging and locomotion strategies according to microhabitat differences (e.g., salinity and water flow velocity) in the Tarim River Basin, while the conserved eye diameter implies functional constraints of visual perception in the relatively turbid saline–alkaline water environment [22]. Notably, the elongated intestinal tract and significantly increased villus height (~210 μm) are the core histological adaptive features of this species: the expanded mucosal surface area effectively improves the absorption efficiency of nutrients and inorganic ions, which not only alleviates nutritional limitation in saline–alkaline water but also provides a structural basis for maintaining cellular osmotic balance [25,26]. These morphological and histological features are typical adaptive traits of fish in saline–alkaline environments and are functionally matched to osmotic regulation and nutrient absorption under saline–alkaline stress.

The significant differences in microbial community structure and diversity between rearing water (ST) and gut contents (TY) fully confirmed the strong host-mediated filtering effect of the *T. yarkandensis* gut on environmental microorganisms, a universal assembly rule of fish gut microbiota verified by numerous studies [27,28]. The drastic reduction in taxonomic richness from ST to TY and only eight shared amplicon sequence variants (ASVs) between the two habitats indicated that the intestinal microenvironment of this species acts as a stringent selective sieve, and this filtering effect is more pronounced than that reported in other saline–alkaline-adapted fish such as *Mugil cephalus* [29] and *Carassius auratus* [20]. This strong selectivity is mainly driven by the unique physicochemical characteristics of the fish gut (anaerobic environment, specific pH, and intestinal mucus composition) and host immune factors, which only allow microbial taxa adapted to the intestinal niche to colonize and reproduce. At the phylum level, the dominance of Proteobacteria in both ST and TY is attributed to its strong adaptability to various extreme environments and diverse metabolic functions; the significant enrichment of *Bacteroidota* and *Firmicutes* in TY is a typical characteristic of fish gut microbiota associated with nutrient metabolism and osmotic stress resistance [30], as these two phyla are widely involved in the degradation of organic matter and synthesis of short-chain fatty acids, providing energy for the host and enhancing its tolerance to saline–alkaline stress. At the genus level, the dominance of *Aurantimicrobium* and *Aestuariivirga* in the gut is a species-specific adaptive feature of *T. yarkandensis*: these taxa have been confirmed to be closely associated with osmoregulation and nutrient absorption of aquatic organisms in saline environments, which is highly consistent with the adaptive needs of this species in the Tarim River Basin, reflecting the long-term co-evolution between the host and its gut microbiota.

The functional differentiation between ST and TY microbial communities showed clear habitat-specific specialization and inter-habitat functional complementarity, which is the key to the stable operation of the “water–gut–host” ecological system in saline–alkaline habitats [30]. PICRUSt2-based KEGG functional prediction showed that ST microbial communities were enriched in environmentally relevant pathways such as xenobiotics biodegradation and starch and sucrose metabolism, which is an adaptive response of saline–alkaline water microorganisms to complex organic pollutants and limited carbohydrate resources, and these pathways not only maintain the material cycling of the aquatic environment but also provide a potential nutrient source for the host. In contrast, TY microbial communities exhibited heightened activity in genetic information processing pathways and significant enrichment of unsaturated fatty acid biosynthesis and ABC transporter pathways, which are directly linked to the host’s saline–alkaline adaptation. Unsaturated fatty acid biosynthesis is critical for maintaining the integrity and fluidity of intestinal epithelial cell membranes under osmotic stress, while ABC transporters mediate the active transport of nutrients and inorganic ions across the cell membrane, directly participating in the host’s osmotic regulation and nutrient assimilation. This functional differentiation verifies the core role of the gut microbiota as the host’s “second genome” [31], and the functional complementarity between water and gut microorganisms forms a stable microecological system that jointly supports the survival of *T. yarkandensis* in extreme saline–alkaline habitats.

Co-occurrence network and topological characteristic analysis revealed the habitat-adaptive structural characteristics of ST and TY microbial communities and identified the core regulatory taxa maintaining community stability. The higher Degree and Eigenvector Centrality of ST nodes indicated that the water microbial community has a more complex interspecific association network, which is an adaptive response to the unstable and heterogeneous saline–alkaline water environment (e.g., frequent fluctuations in salinity and pH). In contrast, the higher Betweenness Centrality of TY nodes suggested that core gut microbial taxa act as critical “bridge nodes”, mediating the material and information exchange between different taxa and maintaining the stability of the gut microbiota in the relatively closed intestinal environment. The identification of *Polynucleobacter* (ST) and *Aurantimicrobium* (TY) as dominant hub nodes confirmed that these taxa are the key to maintaining the microbial network structure, and their high connectivity ensures the functional stability of the microbial community when facing environmental fluctuations [32]. Notably, both ST and TY microbial co-occurrence networks showed typical scale-free topological characteristics (R^2^ > 0.8), which endow the microbial communities with strong disturbance resistance—the loss of ordinary low-degree taxa will not destroy the overall structure and function of the network—and this structural feature is an important evolutionary strategy for the microbial communities of *T. yarkandensis* to adapt to the fluctuating saline–alkaline water environment of the Tarim River Basin.

Ecological niche and community assembly process analyses further clarified the ecological driving mechanism of the structural and functional differentiation of ST and TY microbial communities [33]. ST microbial taxa exhibited broader niche breadth and higher niche overlap, reflecting the extensive utilization of environmental resources and intense interspecific competition in free-living aquatic microbial communities; meanwhile TY microbial taxa showed narrow niche breadth and low niche overlap, a typical host-mediated niche specialization pattern [33], which is conducive to reducing interspecific competition in the intestinal environment and improving the utilization efficiency of specific nutrients, thus better serving the host’s physiological metabolism. The divergent assembly processes of the two microbial communities are the direct cause of their niche differentiation: stochastic processes (NST = 0.71) dominated the assembly of ST microbial communities, which is related to the frequent environmental fluctuations in saline–alkaline water bodies, where random factors such as microbial diffusion and ecological drift play a major role [34]; deterministic processes (NST = 0.32) driven by host selection dominated the assembly of TY microbial communities, which is consistent with the assembly rules of most fish gut microbiota reported in previous studies [34]. The consistency between core contributing taxa and dominant taxa/hub nodes of the two communities indicated that these taxa are not only the core of community structure but also the key driving force of community assembly and niche differentiation, and their specific ecological functions are the basis for the adaptive evolution of microbial communities to different habitats. Quantitative correlation analysis showed that the relative abundance of core gut genera Aurantimicrobium and Aestuariivirga was significantly positively correlated with intestinal villus height, body length and villus width (*p* < 0.05). The Mantel test further confirmed a significant overall correlation between gut microbial community composition and host phenotypic and histological traits (r = 0.62, *p* = 0.003).

The synergistic adaptation between the host *T. yarkandensis* and its associated microbiota is the core strategy for the species to cope with saline–alkaline stress, which conforms to the hologenome theory of evolution [13]. On the one hand, the host’s morphological and intestinal histological adaptive traits provide a stable and suitable ecological niche for the colonization and reproduction of specific gut microbial taxa: the elongated intestinal tract and expanded mucosal surface area increase the attachment sites of microorganisms, and the specific intestinal physicochemical environment screens environmental microorganisms to form a host-specific gut microbiota. On the other hand, the gut microbiota with a specialized structure and function feeds back to the host’s physiological metabolism: core taxa and their enriched functional pathways directly participate in the host’s nutrient absorption and osmotic regulation, and the stable microbial network structure ensures the continuous provision of these functional services, effectively compensating for the host’s physiological pressure under saline–alkaline stress. In addition, the water microbial community, as the main source of the gut microbiota, provides a continuous microbial pool for the host, and the host’s stringent filtering effect ensures that only microbial taxa matching adaptive needs are retained, forming a closed “environment–microbiota–host” synergistic adaptation system. This system is the result of long-term co-evolution between *T. yarkandensis* and its associated microorganisms in the saline–alkaline environment [13] and is the key to the species’ successful colonization and survival in the extreme aquatic ecosystem of the Tarim River Basin.

This study has several unavoidable limitations that need to be acknowledged, and these limitations also point out the direction for future research. First, the static sampling method with a limited number of biological replicates cannot reflect the temporal and spatial dynamic changes in host phenotypic traits and microbial communities with seasonal and hydrological changes (e.g., salinity and water flow) in the saline–alkaline water body. Second, the functional potential of microbial communities was only predicted by PICRUSt2, and no in vitro culture or functional verification experiments were carried out for core taxa, which limits the direct confirmation of their functional role in the host’s saline–alkaline adaptation. Third, the lack of host transcriptome and metabolome data prevents in-depth multi-omics integrated analysis of the molecular mechanism of host–microbiota interaction.

Future research should focus on the following aspects: (1) expanding the sample size and carrying out long-term dynamic monitoring across seasons and different river basins to reveal the temporal and spatial variation characteristics of the host phenotypic traits and microbial communities of *T. yarkandensis*; (2) conducting functional verification of core microbial taxa through in vitro culture, germ-free fish models and microbiota transplantation experiments to clarify their direct regulatory role in the host’s saline–alkaline adaptation; (3) supplementing the host’s multi-tissue transcriptome and metabolome data and carrying out multi-omics integrated analysis to reveal the molecular interaction mechanism between the host and microbiota at a deeper level. Moreover, detailed morphological research on the digestive and excretory systems should be performed to identify the structural adaptations of *T. yarkandensis* to saline–alkaline habitats. In addition, the research results could be applied to the artificial breeding and germplasm resource protection of *T. yarkandensis*, such as optimizing the breeding water microecological environment to improve the survival rate of the species under artificial saline–alkaline conditions.

## 5. Conclusions

This study elucidates the saline–alkaline adaptation mechanisms of the endemic *T. yarkandensis* in the Tarim River Basin via integrated analyses of morphology, intestinal histology and microbial ecology, finding that this fish has evolved a streamlined body shape, plastic morphological traits and elongated intestinal villi as phenotypic and histological adaptations to saline–alkaline flowing water habitats. The fish gut exerts a strong host-mediated filtering effect on environmental microorganisms, leading to significant structural, compositional and functional differentiation between rearing water and gut microbial communities, with the gut-enriching species-specific core taxa (*Aurantimicrobium*, *Aestuariivirga*) and functional pathways closely related to host osmoregulation and nutrient absorption. Additionally, water and gut microbial communities exhibit distinct habitat-adaptive co-occurrence network characteristics and assembly processes, with stochastic processes dominating water microbial assembly and host-driven deterministic processes governing gut microbial assembly. Collectively, the synergistic adaptation between the host’s phenotypic and histological traits and the specialized structure and function of the gut microbiota forms a stable environment–microbiota–host adaptive system, which is the core strategy for *T. yarkandensis* to survive in extreme saline–alkaline habitats. This research not only fills the gap in the integrated study of *T. yarkandensis*’ adaptive traits but also provides important theoretical support for the conservation of this rare endemic fish and the research on environmental adaptation mechanisms of plateau saline–alkaline fish.

## Figures and Tables

**Table 1:** Sequencing data and species composition analysis of environmental and gut content diversity in water bodies.

Sample	Raw Reads	Filtered	Denoised	Merged	Non-Chimeric	Non-Singleton	Order	Family	Genus	Species
ST1	102,532	96,685	95,526	92,996	89,775	89,717	26	113	240	333
ST2	82,833	77,636	75,753	70,402	61,715	61,567	29	194	269	389
ST3	119,414	111,910	109,735	103,831	92,454	92,307	34	206	299	414
TY1	94,830	88,824	88,771	88,635	87,161	87,160	0	4	6	12
TY2	106,736	100,750	99,497	95,591	80,626	80,610	8	34	74	44
TY3	112,151	105,159	104,551	103,244	93,488	93,487	2	7	22	21

## Data Availability

The original contributions presented in this study are included in the article. Further inquiries can be directed to the corresponding author.
